# H3K4 dimethylation at *FosB* promoter in the striatum of chronic stressed rats promotes morphine-induced conditioned place preference

**DOI:** 10.1371/journal.pone.0221506

**Published:** 2019-08-23

**Authors:** Minghui Chen, Xiaojie Zhang, Wei Hao

**Affiliations:** 1 Department of Psychiatry, The Second Xiangya Hospital, Central South University, Changsha, Hunan, China; 2 Mental Health Institute of Central South University, Changsha, Hunan, China; 3 National Clinical Research Center for Mental Disorders, Changsha, Hunan, China; 4 National Technology Institute on Mental Disorders, Changsha, Hunan, China; 5 Hunan Key Laboratory of Psychiatry and Mental Health, Changsha, Hunan, China; Harvard Medical School, UNITED STATES

## Abstract

Expression of *FosB* gene in striatum is essential in addiction establishment. Activated glucocorticoid receptors (GRs) induce *FosB* gene expression in response to stressor. Therefore, elevation of *FosB* expression in striatum serves as one mechanism by which stress increases risk for addiction. In this study, adult male Sprague-Dawley rats were used to investigate whether chronic stress result in histone modifications at *FosB* gene promoter in striatum and how these histone modifications affect *FosB* expression and the establishment of addiction behavior after administration of drugs of abuse. Animals were randomly assigned to three groups: Electric foot shock (EFS) group received 7-day EFS to induce chronic stress; electric foot shock plus mifepristone (EFS + Mif) group were injected with mifepristone, a nonspecific GRs antagonist, before EFS; control group did not receive any EFS. All groups then received 2-day conditioned place preference (CPP) training with morphine (5 mg/kg body weight) to test vulnerability to drug addiction. Before and after morphine administration, *FosB* mRNA in striatum was quantified by real-time RT-PCR. Levels of histone H3/H4 acetylation and histone H3K4 dimethylation at *FosB* promoter in striatum after morphine administration were measured by using chromatin immunoprecipitation (ChIP) plus real-time PCR. EFS group had stronger place preference to morphine and had significantly higher level of *FosB* mRNA in striatum than the other two groups. H3K4 dimethylation was 2.6-fold higher in EFS group than control group, while no statistical difference in H3/H4 acetylation. Mifepristone administration before EFS decreased histone H3K4 dimethylation and *FosB* mRNA in striatum, and also diminished morphine-induced conditioned place preference. Altogether, increased level of H3K4 dimethylation at *FosB* promoter in striatum is partially dependent on the activation of GR and responsible for the elevated level of morphine-induced *FosB* mRNA in chronic stressed animals.

## Introduction

Stress increasing the risk of drug addiction has been observed in animal models as well as humans [1–7]. Hypothalamic-pituitary-adrenal (HPA) axis, which is activated by stressors and abusive drugs, is important in establishment [8] and relapse [9] of addiction. Repeated stress exposure impairs the negative feedback of HPA axis, resulting in a long-lasting increase in glucocorticoid secretion [7]. Glucocorticoid hormones have been shown to potentiate rewarding effects of morphine [10–12] as well as other psychostimulant drugs [13–18]. Mifepristone, a non-specific blocker of glucocorticoid receptors (GRs), reduces locomotor response [19] and conditioned place preference (CPP) [20] to morphine. Dopaminergic transmission of the mesolimbic pathway, which links the ventral tegmental area in the midbrain to the nucleus accumbens (NAc) located in the ventral part of the striatum, is important in mediating the addictive properties of drugs of abuse [21, 22]. Selective deletion of GRs in dopaminergic receptive neurons of NAc attenuates cocaine self-administration [23] as well as cocaine-induced behavioral sensitization and CPP [24]. Therefore, striatum is one key site in which stress mediates abusive drug-induced effects [6].

*FosB* in striatum is a major neural substrate for the rewarding effects of drugs of abuse as well as chronic stress [25–27]. As a transcription factor, *FosB* mediates some of drug-induced changes in gene expression and behavior [28, 29]. Persistently elevated corticosterone induces *FosB* expression in brain [30]. Drugs of abuse regulate expression of *FosB* in striatum through histone modifications at *FosB* promoter [31, 32]. Histone modifications (e.g., acetylation, methylation) in turn facilitate drug-induced behavioral and other emotion-related behaviors [33–36]. We therefore hypothesized that histone modifications at *FosB* promoter in striatum serve as a mechanism by which chronic stress increases the risk for morphine addiction.

Our data confirmed that after chronic exposure to electric foot shock (EFS) rats showed more morphine-induced CPP, which is partially inhibited by GRs antagonist mifepristone. Morphine induced more *FosB* mRNA in striatum in chronic-stressed animals than non-stressed animals, and mifepristone diminished the *FosB* expression in chronic-stressed animals. Using chromatin immunoprecipitation (ChIP) assays, we found that histone H3K4 dimethylation but not histone acetylation at *FosB* gene promoter in striatum was increased by about 2.6 fold in chronic stressed animals and partially inhibited by mifepristone. Increased H3K4 dimethylation at *FosB* promoter in striatum can be a mechanism for morphine-induced *FosB* elevation in chronic stressed animals.

## Materials and methods

### Animal care and use

**Fig 1:**
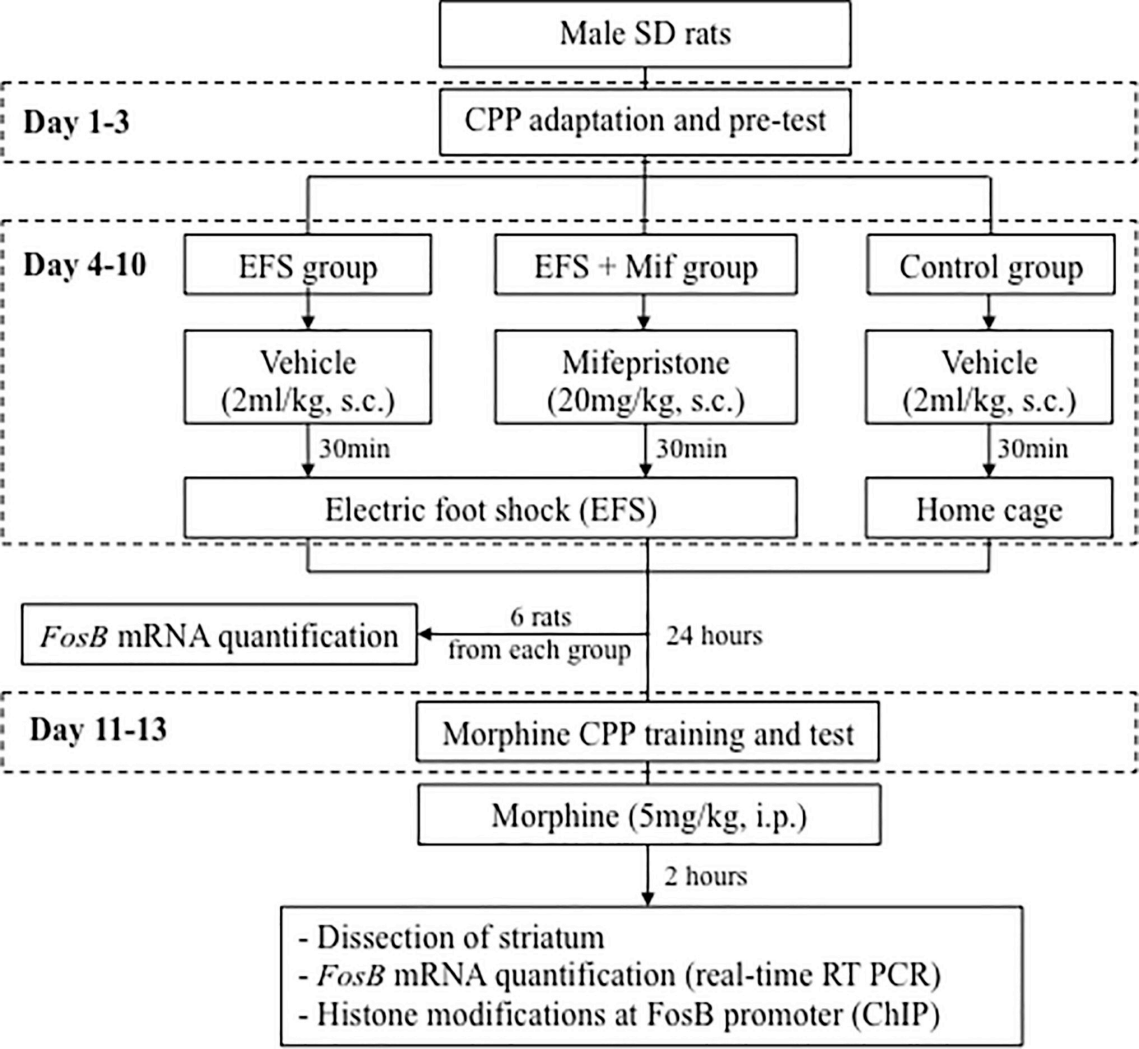
Experimental design.

Male Sprague-Dawley rats (inbred strain; 180–220 g in weight; Animal Center, the Second Xiangya Hospital, Changsha, China) were used for experiments. The animal care and experimental protocols were approved by Animal Care and Use Committee of the second Xiangya Hospital of Central South University. Animals were group-housed (3–4 per cage) with water and food available. The established animal houses having a 12-h light/dark cycle and humidity- and temperature-regulated (25°C) environment. CPP and EFS were performed during the day (i.e., 8 a.m.– 8 p.m.). After behavioral tests (see Fig 1), rats were anesthetized with phentobarbital sodium (40 mg/kg, i.p., Sigma-Aldrich, St. Louis, USA). When being completely anesthetized (i.e., no response to painful stimulus), rats were sacrificed by cervical dislocation.

### Behavioral studies

#### CPP pre-test

The apparatus of CPP (Anilab, Ningbo, China) were consisted of black and white chamber (60 cm × 30 cm × 30 cm) with distinct floor texture (smooth floor in the black and rough floor in the white chamber). The boxes were placed under conditions of dim illumination (40 lux) and masking white noise.

CPP training and test were performed as described previously [37]. Briefly, animals (n = 54) were allowed to adapt to CPP apparatus 60 min per day for 2 days. Their preferences to black or white chamber were examined for 900 s (Day 3, Fig 1) by allowing them move freely in CPP box and recording the time that they spent in each chamber. The less preferred chamber was assigned as morphine-paired chamber and the other one was assigned as saline-paired chamber. The time that rats spent in the less preferred chamber was defined as CPP pre-test. Then, the animals were randomly divided into 3 groups (n = 18 per group).

#### Chronic stress exposure

Chronic stress exposure was performed in a fear conditioning box (Anilab, Ningbo, China) with dimensions of 30 cm × 30 cm × 30 cm. The floor of the box was consisted of steel rods (5 mm in diameter; 15 mm apart), through which the electric current was delivered. The box was placed in a dim room, different from the room where CPP training occurred.

Electric foot shock (EFS) is a well-developed protocol to establish animal model of stress [38–40]. Circulation glucocorticoids level elevated after EFS [16, 41, 42]. To test whether GRs involved in chronic stress-induced morphine CPP, we injected electric foot shock plus mifepristone (EFS + Mif) group with mifepristone (20mg/kg of body weight, Sigma-Aldrich, St. Louis, USA) subcutaneously. Foot shock (EFS) group and control group were injected with vehicle (20% DMSO in saline, 1ml/kg of body weight). Fifteen-minutes after injections, EFS and EFS + Mif groups were then received EFS. Control group stayed in their home cage after injection, so they were neither received foot shock nor brought to the foot shock-related environment.

The foot shock procedure was performed continuously for 7 days (Day 4–10, Fig 1) at a random time during the day. Animals were placed on steel rods. Unpredictable foot shocks of 2 mA intensity were delivered to the grid floor and the current was pulsating with a phase duration of 3 s. Total shock duration was 180 s in a 900 s session (the time between two shocks was ranged from 9 to15 s). The apparatus was cleaned and wiped with 70% ethanol after foot shock of each animal.

#### CPP training

On day 11 and 12, all of the animals received CPP training. Rats were injected with a small dose of morphine hydrochloride (5 mg/kg body weight; Shengyang Pharmaceutical Ltd., China) or saline of the same volume intraperitoneally, and were limited in morphine-paired chamber or saline-paired chamber for 60 min respectively. CPP training was carried out during the 12-hour light phase. There was at least 6 hours between morphine and saline injection. Half of the rats received morphine in the morning and half received morphine in the afternoon. On the next day, the procedure was reversed.

#### CPP testing

After 24 h following the last conditioning pairing, the animals were injected with saline and allowed to enter the black and white chamber freely for 900 s. Movement of animals were tracked and recorded by a camera. The total time that rats spent in each side were calculated by a tracking software (Anilab). The time spent in the morphine-paired chamber was defined as CPP test. CPP score was defined as the result from subtracting CPP pre-test from CPP test.

### Quantitation of *FosB* mRNA by RT-PCR

Six rats from each group were sacrificed 24 hours after the last chronic stress exposure. After CPP test, all of the other animals were injected with morphine (5 mg/kg body weight) and sacrificed within 2 hours. The whole striatum of each rat were collected for RNA quantification or ChIP. RNA was extracted using Trizol reagent (Invitrogen, Carlsbad, CA) and precipitated with isopropanol. mRNA was reverse transcribed to cDNA using a PrimeScript RT reagent kit (TaKaRa Bio, Japan) and PCR system 9700 (Applied Biosystem, Foster city, CA). The amount of cDNA was also quantified using real-time PCR with SYBR green (TaKaRa Bio, Japan). cDNA of *FosB* gene was amplified by using the following primers 5’- GTGAGAGATTTGCCAGGGTC -3’(forward) and 5’- AGAGAGAAGCCGTCAGGTTG -3’ (reverse). GAPDH (5’- AGGTCGGTGTGAACGGATTTG -3’ (forward) and 5’-TGTAGACCATGTAGTTGAGGTCA -3’ (Reverse)), a house-keeping gene, was used as an internal reference for normalization. Relative copy numbers of *FosB* or GAPDH mRNA in each sample were calculated using the standard curve method as described in Rotor-Gene 3000 operator manual (Corbett, Australia). Fold changes of mRNA were calculated by normalizing to *FosB* mRNA levels of control group before morphine injection. For each sample, PCR reactions were repeated at least two independent times.

### Quantitation of histone modifications at *FosB* promoter using Chromatine Immunoprecipitation (ChIP) plus real-time PCR

ChIP was performed following the protocol of ChIP kit (Upstate Biotechnology, Billerica, MA). The whole striatum, was removed from rats by gross dissection, cut into 1 mm-sized pieces, and immediately crosslinked in 1% formaldehyde for 10 min at 37°C. The crosslinking reaction was stopped by adding glycine to a final concentration of 0.125 M for 12 min at 4°C. The tissue was washed four times in ice cold PBS containing proteinase inhibitors (1 mM PMSF, 1 μg/ml aprotinin, and 1 μg/ml pepstatin A) and then frozen at -70°C. Fixed striatum was resuspend in 400 μl SDS lysis buffer (Upstate) and incubate for 10 min on ice. Next, the extracted chromatin was sheared to 300–1000 bp using the Sonic Dismembrator Model 100 (Fisher, Hampton, NH). Each sample was sonicated eight times on ice, 15 sec each, at maximum power. ChIP assays were performed to measure the levels of histone acetylation or methylation at various promoter regions. Equal amounts of chromatin lysate (55 μg) were diluted with ChIP dilution buffer (Upstate) to a final volume of 1.0 ml. Eighty microliters of the pre-immunoprecipitated lysate were saved as “input” for later normalization.

The chromatin solution was pre-cleared with 37.5 μl salmon protein A-agarose/Salmon sperm DNA (50% slurry) (Upstate) for 30 min at 4°C with agitation. It was then immunoprecipitated overnight at 4°C with 2 μg of anti-acetyl-histone H3 (Lys9) (Upstate) or 2 μg of Di-Methyl-Histone H3 (Lys4) Antibody (Cell Signaling Technology). For a negative control, samples were immunoprecipitated with no-antibody. After immunoprecipitation, the antibody/DNA–histone complex was collected with 30 μl of protein A agarose/ salmon sperm DNA (50% Slurry) for 1 hour at 4°C with rotation. The beads were sequentially washed once with low salt (Upstate), high salt (Upstate), and LiCl (Upstate) immune complex wash buffer and washed twice with TE buffers (Upstate). The DNA–histone complex was then eluted from the agarose with 500 μl of freshly prepared elution buffer (0.1 M NaHCO_3_ and 1%SDS). DNA and histones were dissociated at 65°C for 4 hr under high salt conditions. Proteins were digested using proteinase K treatment for 1hr at 45°C. The DNA, associated with acetylated or methylated histones, was extracted with phenol/chlorophorm/isoamyl alcohol, precipitated with 100% ethanol, and finally resuspended in 20 μl of PCR grade water.

Levels of specific histone modifications at each gene promoter of interest were determined by measuring the amount of acetylated or methylated histone associated DNA by quantitative real-time PCR. Specific primers were designed to amplify proximal promoter regions, less than 200 bp long. For *FosB*, the primers 5’- GGGAAGGGAGAGTTCGGG -3’ (forward) and 5’- GGCCTCCAAGAAGAAGAAAAAGA -3’ (reverse) amplified a region 94 bp upstream of the start codon. Glyceraldehyde-3-phosphate dehydrogenase (GAPDH) ((5’- CGTAGCTCAGGCCTCTGCGCCCTT -3’ (forward) and 5’- CTGGCACTGCACAAGAAGATGCGGCTG -3’ (reverse)), was used as controls. Input and immunoprecipitated DNA amplification reactions were run in triplicate in the presence of SYBRGreen (DRR041A, TaKaRa). Ct values from each sample were obtained using the Rotor Gene 3000 software. Relative quantification of template was performed as described previously by Nadia M. Tsankova et al (2004). Briefly, a ΔCt value representing the difference between control Ct and experimental Ct (EFS or EFS + Mif) was calculated, using the formula: ΔCt = (N_exp_-N_avecontrol_)×Ct_avecontrol_. N is the normalized Ct value of immunoprecipitated DNA sample by using anti-H3 acetylation, anti-H4 acetylation or anti-dimethylated H3K4 antibody [N = Ct(IP)/Ct(Input)]. N_exp_ is the mean of N value for EFS or EFS + Mif group. N_avecontrol_ is the average of N value for the control, and Ct_avecontrol_ is the mean of Ct value for the control. Fold differences (EFS or EFS + Mif ChIP relative to control ChIP) were then determined by raising 2 to the ΔCt power. Each PCR reaction, run in triplicate for each brain sample, was repeated at least two independent times.

### Statistical analysis

Data were analyzed using GraphPad Prism 4 (La Jolla, CA). Results are presented as the mean ± SEM. Paired Student’s *t* tests were performed to determine the changes within a group. One-way ANOVA tests were performed to determined difference among three groups. If not specified, unpaired Student's *t* tests were performed between two different experimental groups. The criterion for statistical significance was chosen to be *p* < 0.05.

## Results

### Chronic stress promoted establishment of morphine-induced CPP in rats

**Fig 2:**
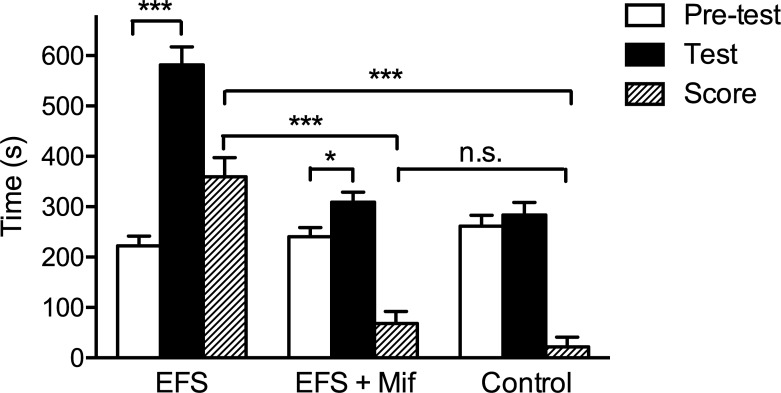
Chronic stress increases morphine CPP, which is partially mediated by GRs. After 7-day EFS, rats received 2-day morphine CPP training. CPP pre-test was the time rats stayed in white chamber before CPP training. All of the rats showed preference to black side, so the white chamber was chosen as morphine-paired side. After 2-day CPP training with morphine, EFS group showed increased preference to morphine-paired chamber, while control group still did not show significant preference to drug-paired side. CPP score is the change in time spending in morphine-paired side before and after CPP training. EFS group showed the highest CPP score, followed by EFS+Mif, and control group showed the lowest CPP score. Blocking GRs using mifepristone significantly inhibited morphine-induced CPP in chronic stressed rats. Bar graphs represent the mean ± SEM. * *p* < 0.05; *** *p* < 0.0001, n.s. means no significance.

The susceptibility to morphine addiction was evaluated using CPP with a small dose of morphine. Before stress exposure and CPP training, rats were injected with saline, and allowed move freely in the CPP apparatus for 900 s. All of the rats preferred black side (658 ± 11 s, n = 36, paired *t*-test: *p* <0.0001), so the white side was chosen to be morphine-paired side to minimize the effect of natural preference on morphine CPP training. CPP pre-test and CPP test were defined as the time spent in white chamber before and after CPP training. CPP score was resulted from subtracting CPP pre-test from CPP test. Rats were divided into three groups randomly, and receive stress exposure and morphine CPP training, as described in the methods (Fig 1). No statistical differences in pre-test among three groups (Fig 2, EFS group: 222 ± 19 s, n = 12; EFS + Mif group: 241 ± 18 s, n = 12; Control: 262 ± 21 s, n = 12, one-way ANOVA test: *p* = 0.37). After 2-day morphine conditioning, animals received a CPP test after saline injection. CPP test showed that EFS group spent significant longer time in the morphine-paired chamber (582 ± 36 s, n = 12) than both EFS + Mif group (309 ± 20 s, n = 12, *p* < 0.0001) and control group (283 ± 25 s, n = 12, *p* < 0.0001). There was no statistical significance in CPP test between EFS + Mif group and control group (*p* = 0.43). By comparing with their pre-test results, we found that both EFS group (Fig 2, 582 ± 36 s, n = 12, paired *t*-test: *p* < 0.0001) and EFS + Mif group (Fig 2, 309 ± 20 s, n = 12, paired *t*-test: *p* = 0.0165) established morphine-induced CPP, but control group (Fig 2, 283 ± 25 s, n = 12, paired *t*-test: *p* = 0.2893) did not show increased preference to morphine-paired chamber. Using CPP score to evaluate morphine addiction, we found that EFS group (359 ± 38 s, n = 12) showed more preference to morphine-paired side than control group (22 ± 20 s, n = 12, *p* < 0.0001). The results indicate that chronic stress facilitates establishment of morphine-induced CPP. Administration of GR antagonist mifepristone before stress exposure significantly inhibited morphine-induced CPP in chronic stressed animals (68 ± 24 s, n = 12; compared with EFS group: *p* < 0.0001; compared with control: *p* = 0.1496), suggesting that activation of GRs mediated chronic stress induced morphine addiction.

### Morphine changed the transcriptional regulation of *FosB* in striatum of chronic-stressed rats

**Fig 3:**
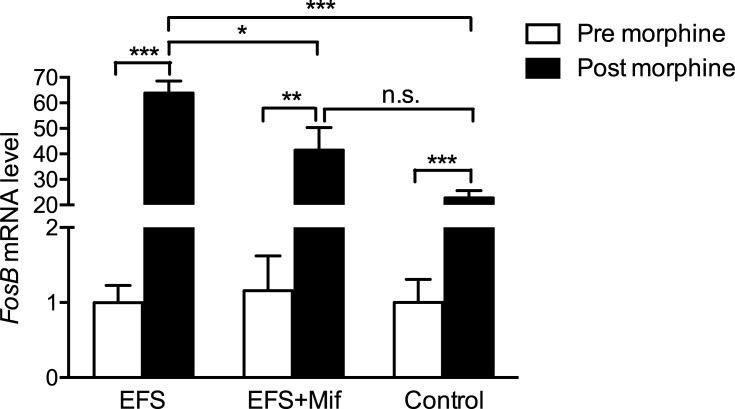
Previous chronic stress enhanced morphine-induced *FosB* expression in striatum, which was partially dependent on GRs. *FosB* mRNA level in striatum were quantified by real-time RT-PCR. Before morphine administration, there was no statistical difference in levels of *FosB* mRNA in striatum either between EFS group and control or between EFS + Mif and control. Within 2 hours of the last morphine injection, *FosB* mRNA in striatum was hyperregulated significantly in all of the three groups. Furthermore, EFS group had the highest level of *FosB* mRNA. Blocking GRs using mifepristone attenuated morphine-induced *FosB* expression in EFS rats. Bar graphs represent the mean ± SEM. * *p* < 0.05, ** *p* < 0.01, *** *p* < 0.0001, n.s. means no significance.

*FosB* expression is induced in striatum by morphine administration [25] as well as chronic stress [43–45]. To test whether chronic-stressed animals express more *FosB* in striatum after morphine administration and whether GRs are involved in regulation of *FosB* expression, *FosB* mRNA in striatum was quantified in rats before morphine administration and within 2 hours of morphine administration using real-time RT-PCR. Before morphine administration (i.e., 24 hours after chronic stress), there was no statistical significance among EFS group (Fig 3, 0.997 ± 0.230, n = 6), EFS + Mif group (1.158 ± 0.462, n = 5,) and control group (1.000 ± 0.307, n = 6, one-way ANOVA: *p* = 0.93). Immediately after morphine administration (i.e., within 2 hours of the last morphine injection), *FosB* mRNA in striatum was upregulated compared to that before morphine administration in all three groups (Fig 3, EFS group: *p* < 0.0001; EFS + Mif group: *p* = 0.0018; control: *p* < 0.0001). Interestingly, *FosB* mRNA in striatum of EFS group was hyperregulated by 2.8 folds (Fig 3, 63.9 ± 4.7 folds to baseline, n = 6) compared to control (22.8 ± 2.8 folds to baseline, n = 6, *p* < 0.0001). Injection of nonspecific GR antagonist mifepristone before EFS inhibited about 45% of morphine-induced *FosB* expression in EFS rats (Fig 3, EFS + Mif: 41.5 ± 8.8 fold, n = 5; compared with EFS group: *p* = 0.043; compared with control group: *p* = 0.056).

### Stress exposure enhanced histone H3K4 dimetylation at the *FosB* promoter

To determine whether histone modifications involved in morphine-induced *FosB* expression in chronic stressed animals, we measured histone H3K4 dimethylation and H3/H4 acetylation at *FosB* promoter in striatum using ChIP. To control for the specificity of antibody binding, we immunoprecipitated chromatin samples with nonimmune IgG, which precipitated negligible level of the gene studied. To ensure that our technique would allow us to measure the levels of histone modifications only at transcriptional levels in the genome where respective histone modulation is present *in vivo*, we also measured levels of H3K4 dimethylation, histone H3 acteylation and histone H4 acteylation in striatum at the promoters of the GAPDH gene. GAPDH is one of house keeping genes. We assumed that the expression levels of GAPDH remained the same in each group. As expected, no significant changes were found in levels of H3K4 dimethylation (1.000 ± 0.006 fold, n = 5; EFS + Mif: 0.997 ± 0.005 fold, n = 5; Control: 1.000 ± 0.009 fold, n = 5, one-way ANOVA test: p = 0.93), histone H3 acteylation (EFS: 1.02 ± 0.237 fold, n = 5; Control: 1.02 ± 0.152 fold, n = 5; *p* = 0.983) and histone H4 acteylation (EFS: 0.95 ± 0.157 fold, n = 5; Control: 1.02 ± 0.134 fold, n = 5; *p* = 0.732) at the GAPDH gene promoter in striatum. These findings indicate that the observed changes in histone modifications at the *FosB* promoter were not global, but were limited to gene with expression that varies as a result of stress exposure.

**Fig 4:**
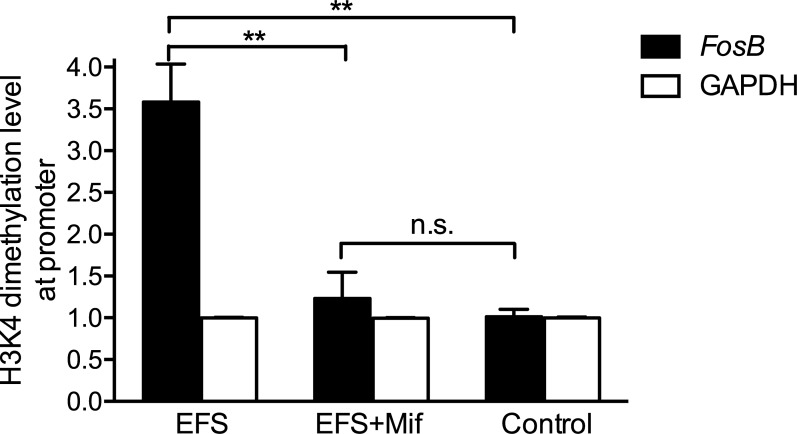
Histone H3K4 dimethylation at *FosB* promoter in striatum increased in chronic stressed rats. ChIP was performed with anti-dimethylated H3K4 antibodies, and levels of *FosB* promoter in the immunoprecipitates were measured by real-time PCR. Histone H3K4 dimethylation at *FosB* promoter (black) and GAPDH promoter (white) were detected in EFS group, EFS + Mif group and control group. There was no significant difference in H3K4 dimethylation at the promoter of house keeping gene GAPDH in three groups. EFS group showed highest level of H3K4 dimethylation at *FosB* promoter. Mifepristone inhibited this histone modification in chronic stressed animals. Bar graphs represent the mean ± SEM. ** *p* < 0.01, n.s. means no significance.

H3K4 dimethylation at *FosB* promoter in striatum of EFS group (Fig 4, 3.58 ± 0.454 fold, n = 5) were about 2-fold higher than EFS + Mif group (1.23 ± 0.314 fold, n = 5, *p* = 0.003) and 2.6-fold higher than control group (1.01 ± 0.089 fold, n = 4, *p* = 0.002). Injection of nonspecific GR antagonist mifepristone before foot shock diminished more than 90% of chronic stress-related H3K4 dimethylation at *FosB* promoter. There was no significant difference in H3K4 dimethylation at *FosB* promoter between EFS + Mif group and control group (*p* = 0.57). No significance difference was found in levels of H3 acetylation (EFS: 1.04 ± 0.291 fold, n = 5; Control: 1.03 ± 0.112 fold, n = 5; *p* = 0.972) or H4 acetylation (EFS: 0.95 ± 0.107 fold, n = 5; Control: 1.04 ± 0.107 fold, n = 5; *p* = 0.658) at *FosB* promoter between EFS group and control group.

## Discussion

The CPP results in our study confirm that chronic stress increases susceptibility to drugs of abusive [1–6]. The chronic stressed animals in this study established morphine-induced CPP by administrating lower dose of morphine after a shorter training period than unstressed rats reported before [37, 46]. Stress facilitates addiction has also found in animals treated with other types physical stress (e.g., forced swimming stress [47] and restraint stress [48]) or psychological stress (e.g., social defeat stress [49]). Blocking GRs using mifepristone profoundly reduces the motivation of cocaine self-administration [50] and morphine-induced CPP [20]. Consistent with previous studies [11, 12], our results suggest that activation of HPA axis and GRs are significant in early expression of morphine place preference in chronic stressed animals. Inactivation GR gene in the entire brain reduces cocaine self-administration [50]. Selective knockout GR gene in dopamine receptive neuron in NAc reduces chronic stress-induced cocaine self-administration [23]. However, stress was also showed to be inhibitory to acquisition of morphine-induced CPP [51]. These different effects of stress manipulations on place preference conditioning might be explained that the relationship between stress and drug reactivity is not a simple linear function but an inverted U-shaped function [52]. When HPA axis activity is low and increasing to a moderate level, drug reinforcement increases as HPA activity increases, a relationship as we observed using CPP and other studies using self-administration [16]. When further increases in stress reactivity, drug reinforcement decreases as HPA activity increases beyond some moderate level and as it approaches a high level. Based on the inverted U-shape relationship between stress and addiction, our results suggest that the dose of stress we employed in the study is moderate and below the level of stress that would inhibit drug addiction.

*FosB* and its truncated form Δ*FosB* mediate behavioral plasticity in drug addiction by their transcriptional effects [43]. Overexpression of Δ*FosB* in the NAc increased the sensitivity to the rewarding effects of morphine and led to exacerbated physical dependence in mice [53]. Drugs of abuse (e.g., cocaine, opiates and nicotine) as well as chronic stress induce Δ*FosB*, which expresses predominantly in the striatum (NAc and dorsal striatum) [25]. Our results showed that 24 hours after stress termination, chronic stressed animals did not express more *FosB* mRNA in striatum than controls or mifepristone administration group, indicating that chronic stress-induced *FosB* in striatum returns quickly to baseline when stress is terminated. It is consistent with previous *in vivo* and *in vitro* studies that expression of *FosB*, an immediate early gene, returns back to normal within a few hours after the termination of stressors or drugs of abuse [54,55]. Interestingly, morphine induced more *FosB* mRNA in striatum in chronic stressed animals than controls, which suggests substantial changes already exist in the regulators of *FosB* gene in chronic stressed animals before morphine administration. *FosB* is regulated by activated GRs in brain [56]. Corticosterone is involved in regulation of neural *FosB* expression to repeated restraint stress [30]. Consistently, we found that blocking GRs using mifepristone attenuated morphine-induced *FosB* gene expression in chronic stressed rats. GR-mediated *FosB* expression in striatum may serve as one mechanism by which stress increases risk for addiction, as seen in many animal models as well as in humans.

**Fig 5:**
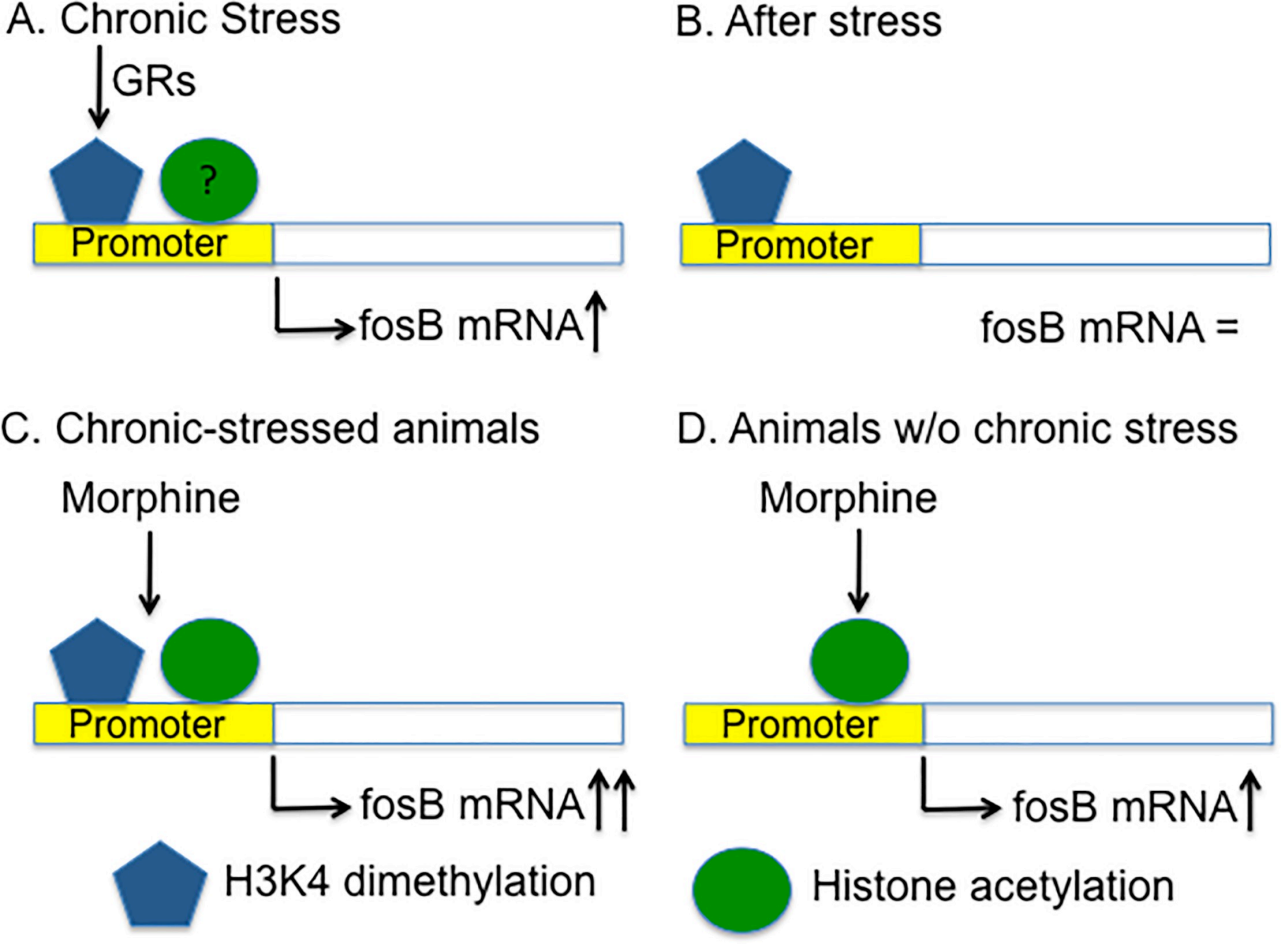
Histone H3K4 dimethylation at *FosB* promoter in the striatum of chronic stressed rats promotes establishment of addiction. (A) Chronic stress induces histone H3K4 dimethylation and may also induce histone acetylation at *FosB* promoter in striatum, which result in the increase of *FosB* expression. H3K4 dimethylation is partially dependent on the activation of GRs. (B) After the termination of chronic stress, histone H3K4 dimethylation at *FosB* promoter still exists, although *FosB* mRNA level return to normal. (C-D) Morphine induces histone acetylation at *FosB* promoter in all of animals. However, preformed H3K4 dimethylation at promoter enables chronic stressed animals transcript more *FosB* mRNA than non-stressed animals when they receive morphine. Higher level of *FosB* in striatum will finally mediate the drug-induced behavioral change.

Although both histone acetylation and histone H3K4 dimethylation promote gene expression [57], we found that only H3K4 dimethylation but not histone acetylation increased at *FosB* promoter in striatum of stressed animals. One explanation for the inconsistent changes of these two activating modifications at *FosB* promoter is that the strong stimulating effect of morphine on *FosB* expression increases histone acetylation at *FosB* promoter substantially in three groups of animals [35], which masks histone acetylation at *FosB* promoter formed during chronic stress. Another possibility is that histone acetylation is a transient modification and cannot retain as long as histone methylation [57], so histone acetylation formed during chronic stress disappear after stress (Fig 5) when *FosB* expression returns to normal. To address this question, the level of histone H3/H4 acetylation and H3K4 dimethylation after chronic stress but before morphine administration in three groups of animals should be measured in future studies. In addition, there are many other modulators of *FosB* transcription. Further studies will be required the address the changes of other types of histone modifications at *FosB* promoter in chronic stressed animals.

Our results showed that blocking GRs using mifepristone decreased H3K4 dimethylations at *FosB* promoter in chronic stressed animals. The inhibitory effect of mifepristone on histone H3K4 dimethylation at *FosB* promoter suggests that GRs interact with histone H3K4 methyltransferase directly or indirectly to regulate *FosB* gene. Previous studies found that GRs regulate histone methylations by interacting with enzymes, such as methyltransferase [58]. GRs are also involved in other types of histone modifications. For example, psychological stress-induced H3 phosphorylation and acetylation in adult gyrus granule neurons involved GRs [59, 60]. Future research will be needed to address the signaling pathway that mediating H3K4 dimethylation. Does H3K4 already be dimethylated during chronic stress or does it occur after the administration of addictive drugs? Does H3K4 dimethylation at promoter is sufficient to activate *FosB* gene or whether it only plays a facilitatory role in *FosB* expression? How long does histone H3K4 dimethylation persist to serve as a potential vulnerability to addiction? Although these questions has not been answered by the present study, we propose that during chronic stress, activation of GRs induces H3K4 dimethylation at *FosB* promoter, which makes *FosB* gene more readily to be turned on when animals are exposed to morphine (Fig 5A–5C). In addition, other types of histone modifications as well as DNA methylations [61, 62] may also be involved in stress-potentiated morphine CPP. Together, our results suggest that chronic stressed individuals possess certain epigenetic modifications on addiction-related genes, which will in turn increase the susceptibility to addictive drugs in the future, even when chronic stress is terminated.

## Conclusions

Increased H3K4 dimethylation at *FosB* promoter in the striatum of chronic stressed rats is partially dependent on the activation of GR, and facilitates morphine-induced *FosB* expression and establishment of morphine CPP.

## Abbreviation

ChIP: chromatin immunoprecipitation
CPP: conditional place preference
EFS: electric foot shock
GAPDH: glyceraldehyde-3-phosphate dehydrogenase
GR: glucocorticoid receptor
H3K4: histone 3 lysine 4
HPA: Hypothalamic-pituitary-adrenal
Mif: mifepristone
NAc: nucleus accumbens
RT-PCR: reverse transcription Polymerase Chain Reaction
